# The language of healthcare worker emotional exhaustion: A linguistic analysis of longitudinal survey

**DOI:** 10.3389/fpsyt.2022.1044378

**Published:** 2022-12-16

**Authors:** Franz F. Belz, Kathryn C. Adair, Joshua Proulx, Allan S. Frankel, J. Bryan Sexton

**Affiliations:** ^1^Duke School of Medicine, Duke University, Durham, NC, United States; ^2^Duke Center for Healthcare Safety and Quality, Duke University Health System, Durham, NC, United States; ^3^Safe and Reliable Healthcare, Evergreen, CO, United States

**Keywords:** burnout, emotional exhaustion, stress, well-being, LIWC, linguistic analyses, healthcare worker (HCW), healthcare quality

## Abstract

**Importance:**

Emotional exhaustion (EE) rates in healthcare workers (HCWs) have reached alarming levels and been linked to worse quality of care. Prior research has shown linguistic characteristics of writing samples can predict mental health disorders. Understanding whether linguistic characteristics are associated with EE could help identify and predict EE.

**Objectives:**

To examine whether linguistic characteristics of HCW writing associate with prior, current, and future EE.

**Design, setting, and participants:**

A large hospital system in the Mid-West had 11,336 HCWs complete annual quality improvement surveys in 2019, and 10,564 HCWs in 2020. Surveys included a measure of EE, an open-ended comment box, and an anonymous identifier enabling HCW responses to be linked across years. Linguistic Inquiry and Word Count (LIWC) software assessed the frequency of one exploratory and eight *a priori* hypothesized linguistic categories in written comments. Analysis of covariance (ANCOVA) assessed associations between these categories and past, present, and future HCW EE adjusting for the word count of comments. Comments with <20 words were excluded.

**Main outcomes and measures:**

The frequency of the linguistic categories (word count, first person singular, first person plural, present focus, past focus, positive emotion, negative emotion, social, power) in HCW comments were examined across EE quartiles.

**Results:**

For the 2019 and 2020 surveys, respondents wrote 3,529 and 3,246 comments, respectively, of which 2,101 and 1,418 comments (103,474 and 85,335 words) contained ≥20 words. Comments using more negative emotion (*p* < 0.001), power (i.e., references relevant to status, dominance, and social hierarchies, e.g., own, order, and allow) words (*p* < 0.0001), and words overall (*p* < 0.001) were associated with higher current and future EE. Using positive emotion words (*p* < 0.001) was associated with lower EE in 2019 (but not 2020). Contrary to hypotheses, using more first person singular (*p* < 0.001) predicted lower current and future EE. Past and present focus, first person plural, and social words did not predict EE. Current EE did not predict future language use.

**Conclusion:**

Five linguistic categories predicted current and subsequent HCW EE. Notably, EE did not predict future language. These linguistic markers suggest a language of EE, offering insights into EE’s etiology, consequences, measurement, and intervention. Future use of these findings could include the ability to identify and support individuals and units at high risk of EE based on their linguistic characteristics.

## 1 Introduction

Emotional exhaustion (EE) in healthcare workers (HCWs) has been linked to higher rates of substance abuse, depression, and suicidal ideation (1–5). EE is also associated with higher rates of medical errors, healthcare-associated infections, and suboptimal patient care (e.g., not answering patients’ questions or not fully discussing treatment options) (6–11). Unfortunately, rates of EE in HCWs have reached concerning levels with 25 to 45% of physicians and 35 to 50% of nurses estimated to have EE (12–15). Recent research suggests that the COVID-19 pandemic has exacerbated EE and mental health issues in HCWs (16, 17). Increasingly, there is interest in signals that predict which groups and work settings are more vulnerable to EE (18, 19). Identifying unobtrusive signals of EE in HCWs could be welcomed by leaders in need of data, and HCWs themselves, who may be unenthusiastic to fill out another burnout survey (20).

Prior research demonstrates that linguistic characteristics of written texts reliably predict the depression level of the writer (21–24). Moreover, the linguistic characteristics of Facebook and Twitter posts have been shown to reliably predict current and future depression, with areas under the curve ranging from 0.67 to 0.89 (25–27). Identifying a pattern of linguistic characteristics for those with EE, or a “language of EE,” could provide a non-invasive method to help health systems identify work settings as potential hot spots for burnout. This pattern could also foster insights into risk factors for EE and opportunities for its prevention (28–30). For example, a workplace shift toward more negative or power-structure-oriented language could signal to administrators a need to recalibrate current management practices.

Psychological research on linguistic categories has revealed compelling linguistic patterns based on personal well-being (22–24, 31) and one’s work culture (21). More specifically, two studies have examined the link between language and EE or burnout. Collectively, they found that greater use of negative emotion (e.g., hurt, ugly, and nasty) words was associated with higher HCW EE (23, 24). Greater use of future tense verbs (e.g., may, will, soon), positive emotions (e.g., love, nice, sweet), references to humans (e.g., adult, baby, boy) and friends (e.g., buddy, neighbor), and assent (e.g., agree, OK, yes) was associated with lower EE (24).

The first study examined how perinatal loss on Italian maternal wards affected the language use of 162 health professionals’ language (21). The authors assessed associations between 56 linguistic categories and the three components of burnout as measured by the Maslach Burnout Inventory (MBI): EE, depersonalization, and personal accomplishment. Although this study advances our understanding of language and burnout, it is limited by its exploratory nature, cross-sectional design, sample size, non-parametric analyses, and increased likelihood of spurious findings due to conducting 168 comparisons.

The second study examined the efficacy of a web-based gratitude letter–writing intervention for improving American healthcare workers’ well-being (20). In a sub-analysis, the study assessed correlations between linguistic categories used in gratitude letters by one) baseline EE (*n* = 1179; cross-sectional design) and change in EE at a 1-week follow-up (*n* = 238; baseline linguistics predicting EE change). The analyses of the five selected linguistic categories is hypothesis-driven and uses larger sample size and parametric analyses. However, the small number of linguistic categories and 1-week follow-up complicated by an 80% dropout rate limits the study’s ability to identify a language of EE. Given the limitations of these studies, the emerging research on a language of EE would benefit from a hypothesis-driven, large-scale, longitudinal assessment of how relevant linguistic categories relate to EE across time.

In comparison to EE, there are several studies on associations between language use and depression. Depressed individuals have been shown to use more words overall, and more first person singular (e.g., I, me, and mine), and present focus (e.g., today, is, now) when describing negative memories. For positive memories, they used more present focus, but fewer words overall, and fewer first person singular and positive words (31). Lastly, in a study of HCW’s capacity to conduct quality improvement activities, called improvement readiness, higher readiness was associated with a lower word count (WC; i.e., number of words), greater use of positive emotions, social words (e.g., mate, talk, and they), and first person plural (e.g., we, us, our; at the level of a trend), and less first person singular, past tense verbs (e.g., ago, did, talked), and negative emotion (including the subcategories of anxiety, anger, and sadness) (21).

The linguistic category of power (i.e., references relevant to status, dominance, and social hierarchies; e.g., own, order, and allow) is of particular interest for EE. Increased references to power may capture HCW frustrations with the medical system and its hierarchies, and thus be associated with EE (32). As social psychologist Susan Fiske identified in her research, attention follows those who control one’s outcomes (33).

Based on prior research linking language to depression, and early findings from studies of HCW writings and EE, we developed a series of hypotheses on associations between linguistic category use and EE. We used a 2-year longitudinal data set and expected associations to emerge cross-sectionally (i.e., within the same year), as well as longitudinally (i.e., EE in year 1 predicting language use in year 2’s comments, and language use in year 1 predicting year 2’s EE). Regarding the specific linguistic categories of interest, we hypothesized HCWs with higher EE would use more words overall and more first person singular, past and present tense verbs, negative emotion, anxiety, anger, and sadness. We hypothesized lower EE would associate with greater use of first person plural, future tense verbs, positive emotions, social words, family, friends, and assent. The lack of prior linguistic evidence associating power words and EE precluded a directional hypothesis, and therefore was considered exploratory.

### 1.1 Objectives

This study examined how the frequency of linguistic categories in HCW comments differed based on their past, current, and future EE.

## 2 Materials and methods

### 2.1 Design and population

This is a longitudinal retrospective study that assesses associations between the frequency of linguistic categories in HCW comments and their prior, future, and current (assessed within the same survey) EE. We used a linked 2019 and 2020 dataset from annual surveys of HCWs in a medium-sized Midwestern health system. Specifically, respondents completed the validated Safety, Communication, Operational, Reliability, and Engagement (SCORE) survey, used for quality improvement of the workplace (34). Participation was voluntary.

The data were collected and anonymized by a survey vendor, Safe and Reliable Healthcare, before they were shared with the first, second, and final authors for analysis and archiving. Safe and Reliable Healthcare provide healthcare systems with assessments of constructs related to patient safety and quality, as well as actionable feedback for targeted improvement. This study was approved by the Institutional Review Board at Duke University Medical Center (Pro00083427).

### 2.2 Materials

#### 2.2.1 SCORE survey

The survey included three main question sections: seven psychometrically validated scales (including EE), ten demographic variables, and an opportunity to leave a comment. An anonymous identifier allowed individuals’ responses to be linked across the 2019 and 2020 surveys. Thus, the comments’ linguistic categories were available for comparisons to EE within the same survey year (i.e., 2019_*EE*_–2019_*comments*_, 2020_*EE*_–2020_*comments*_) and to prior (i.e., 2020_*EE*_–2019_*comments*_) and future years (i.e., 2019_*EE*_–2020_*comments*_).

### 2.3 Demographics

Demographic variables were captured in 2020: age, gender, race, job classification, and whether the employee is a registered nurse and/or a supervisor. Thus, demographic data for 2019 participants was only available if they also completed the survey in 2020. Physicians were not included in this SCORE survey, because they were surveyed separately and at different times.

### 2.4 Emotional exhaustion

Emotional exhaustion was measured with the SCORE survey’s five-item EE derivative of the nine-item MBI EE scale (35). The five-item scale has been demonstrated as reliable and valid (36–38). An example item is *“I feel frustrated by my job”* (34). Response options range from one (disagree strongly) to five (agree strongly). The mean of these five items is then scaled from 0 to 100, with higher scores indicating greater EE (34). Responses with more than two items missing were excluded. For scores missing one to two items, the mean was calculated based on present items. EE scores demonstrated excellent internal consistency with coefficient alphas of α = 0.93 (2019 EE), and α = 0.95 (2020 EE).

### 2.5 Linguistic analyses of comments

At the end of the survey, HCWs were prompted to answer the question: *“Do you have any other comments, questions, or concerns? Please note that while the survey is anonymous and your individual responses above are confidential and never revealed as an individual response, your free text response below will be sent back to the institution verbatim and associated with your work setting. Your institution may distribute the responses back to work setting leaders either verbatim or in summary form.”*

Healthcare worker responses were analyzed by Linguistic Inquiry and Word Count (LIWC) software version 2015 (39). The software references an internal dictionary of 6,400 words and word stems to classify text into 90 output variables (40). Based on the theoretically interesting linguistic themes described earlier, sixteen categories were selected for analysis: Word Count, First Person Singular (e.g., I, me, and mine), First Person Plural, Past Focus (e.g., ago, did, and talked), Present Focus, Future Focus, Positive and Negative Emotion (e.g., hurt, ugly, and nasty), Anxiety, Anger, Sad, Power (e.g., own, order, and allow), Social (e.g., mate, talk, and they), Family, Friends, and Assents (e.g., agree, OK, and yes). More example words are available in Supplementary Appendix Table 1. Word Count (WC) is the number of words in an analyzed text. The other 15 linguistic categories are reported as the percent proportions of words of that category in the text. For example, in the sentence “*I love* working with *my* coauthors to improve *my* paper,” the software documents 30% of words (3 of 10) contain First Person Singular (*I*, *my, and my*) and 10% Positive Emotion (*love*).

Linguistic Inquiry and Word Count proportions are dependent on the denominator, which is the total WC of the text. Thus, proportions in shorter texts (WC ≤ 100) are more variable, yielding less reliable results (41). Since most study comments were shorter than 100 words, linguistic categories with proportions less than 1% were considered unreliable and excluded from analyses. To further improve proportion reliability, linguistic analyses were restricted to comments with WC ≥ 20, which is the conservative, approximate length of a modern day sentence (40, 42). By using this threshold we are reducing the extreme variability exhibited in low word count comments, moreover, we are more likely capturing a fully formed thought or sentence rather than short common phrase, such as “n/a,” “nothing to add,” “nope.”

### 2.6 Statistical analyses

Study hypotheses were assessed with analysis of covariance (ANCOVA). Associations between EE and the 16 linguistic categories were assessed within the same year (2019 and 2020) and across years (2019_*EE*_–2020_*comments*_ and 2020_*EE*_–2019_*comments*_). Linguistic categories with frequency proportions less than 1%, were considered too infrequent to make valid inferences, and were therefore excluded from further analyses (43). *Post hoc* pairwise comparisons then examined the frequency of each linguistic category across the lowest (least exhausted) and highest (most exhausted) quartiles of HCW EE. Since longer comments have been shown to associate with worse mental health and influence the frequency with which a linguistic category is used, analyses of the other 15 linguistic categories controlled for the word count of comments (21, 31). Therefore, word count was used as a covariate for other word categories, but was also analyzed as its own linguistic category variable. Sensitivity analyses additionally controlling for age, gender, and race can be found in the Supplementary Appendix Table 2. Assumptions of linearity were met by natural-log-transforming the categories Present Focus and WC. Due to concerns about multiple comparisons in the current study (*n* = 36), increasing the likelihood of type one error, we calculated a Bonferroni-corrected *p*-value of 0.0014.

## 3 Results

### 3.1 Participants

Overall, 11,336 HCWs responded in 2019 and 10,564 in 2020. Overall, 3,529 surveys contained comments in 2019 and 3,246 in 2020. Filtering for comments with WC ≥ 20 left 2,109 comments for analysis in 2019 and 1,606 in 2020. Demographic data (i.e., age, gender, and race) was available for 929 comments in 2019 and 1,423 in 2020, refer to Table 1. HCWs were mostly in their 40 and 50 s, predominantly female (83.3%), and White (88.3%). For a more detailed demographic breakdown, refer to Supplementary Appendix Table 3.

**TABLE 1:** Respondent demographics for surveys with 20 or more words.

Demographics	2019 comments (WC ≥ 20)	2020 comments (WC ≥ 20)
		
	*N* = 929	*N* = 1,423
Age (μ; 95% CI)	46.7 (45.9, 47.4)	46.0 (45.4, 46.6)
**Gender**		
Female	800 (86.1%)	1,238 (87.0%)
Male	129 (13.9%)	185 (13.0%)
**Race**		
Two or more	11 (1.2%)	13 (0.9%)
American Indian or Alaska Native	7 (0.8%)	7 (0.5%)
Asian	12 (1.3%)	24 (1.7%)
Black	46 (5.0%)	46 (3.2%)
Hispanic/Latino	26 (2.8%)	25 (1.8%)
Pacific Islander or Native Hawaiian	1 (0.1%)	1 (0.1%)
White	826 (88.9%)	1,305 (91.7%)
Other	0 (0%)	1 (0.1%)
Is a registered nurse? (Yes)	290 (31.2%)	402 (28.3%)
Is a supervisor? (No)	857 (92.2%)	1,342 (94.3%)
**Job classification**		
Administrative support workers	178 (19.2%)	247 (17.4%)
Craft workers	3 (0.3%)	2 (0.1%)
Executive/Sr level officials	6 (0.6%)	2 (0.1%)
First/mid-level officials	85 (9.1%)	87 (6.1%)
Professionals	454 (48.9%)	639 (44.9%)
Service workers	97 (10.4%)	153 (10.8%)
Technicians	106 (11.4%)	189 (13.3%)
2019 emotional exhaustion (μ; 95% CI)	56.0 (53.9, 58.1); *N* = 928	52.0 (49.7, 54.2); *N* = 770
2020 emotional exhaustion (μ; 95% CI)	57.0 (55.0, 59.0); *N* = 924	60.2 (58.5, 61.9); *N* = 1,418

In total, 2019 (*N* = 2,109) and 2020 (*N* = 1,606) respondents had comments with a word count (WC) of 20 or more. Demographic data, as reported above, was available for some of these respondents in 2019 (*n* = 929) and 2020 (*n* = 1,423). The continuous variables age and emotional exhaustion (EE) are reported as the mean (with 95% confidence intervals). 2019 and 2020 EE is reported for both 2019 and 2020 comments, as it reflects the EE data available for that year’s set of comments (e.g., 924 EE responses from 2020 for comments with responses ≥20 words in 2019). The categorical variables are reported as the number of respondents (with percent makeup of the demographic category). For EE scores, there are 8 (0.3%) missing responses for 2019 and 5 (0.3%) missing responses for 2020. In 2020, 183 respondents commented without filling out the remaining survey items.

### 3.2 Missing data

In 2020, 260 respondents commented without filling out the remaining survey items. There are 39 (0.3%) missing responses for EE scores in 2019 and 322 (3.0%) missing for 2020. Sensitivity analyses controlling for demographic variables did not meaningfully change the results. See Supplementary Appendix Table 2.

### 3.3 Linguistic category base rates

The seven linguistic categories of Future Focus, Sad, Anger, Anxiety, Assent, Family, and Friends were excluded for having proportions less than 1%, a base rate too low to make valid inferences (43). ANCOVA assessed the associations between EE and the nine remaining linguistic categories: Word Count, First Person Singular, First Person Plural, Positive Emotions, Negative Emotions, Power, Present Focus, Past Focus, and Social.

### 3.4 Outcomes

The mean EE for all survey respondents was 45.3 (95% CI, 44.9–45.8; *n* = 11,284) in 2019, and 44.8 (95% CI: 44.4–45.3; *n* = 10,223) in 2020. Table 1 shows the mean EE increased when filtering for comments with WC ≥ 20, 59.2 (95% CI, 57.9–60.6; *n* = 2,109) for 2019, and 60.2 (95% CI: 58.5–61.9; *n* = 1,603) for 2020. Respondents writing comments of WC ≥ 20 in 2019 and 2020 scored significantly higher (6.7–15.4 points; *p* < 0.01) on EE than the overall cohort. WC for 2019 comments ranged from 20 to 1509 with a median of 63 and mean of 93; comments for 2020 ranged from 20 to 623 with a median of 44 and mean of 61. After log transforming the WC category, there were no outliers. The ranges and sample sizes for the EE quartiles of each comparison are documented in Supplementary Appendix Table 4.

#### 3.4.1 Same-year associations

**FIGURE 1:**
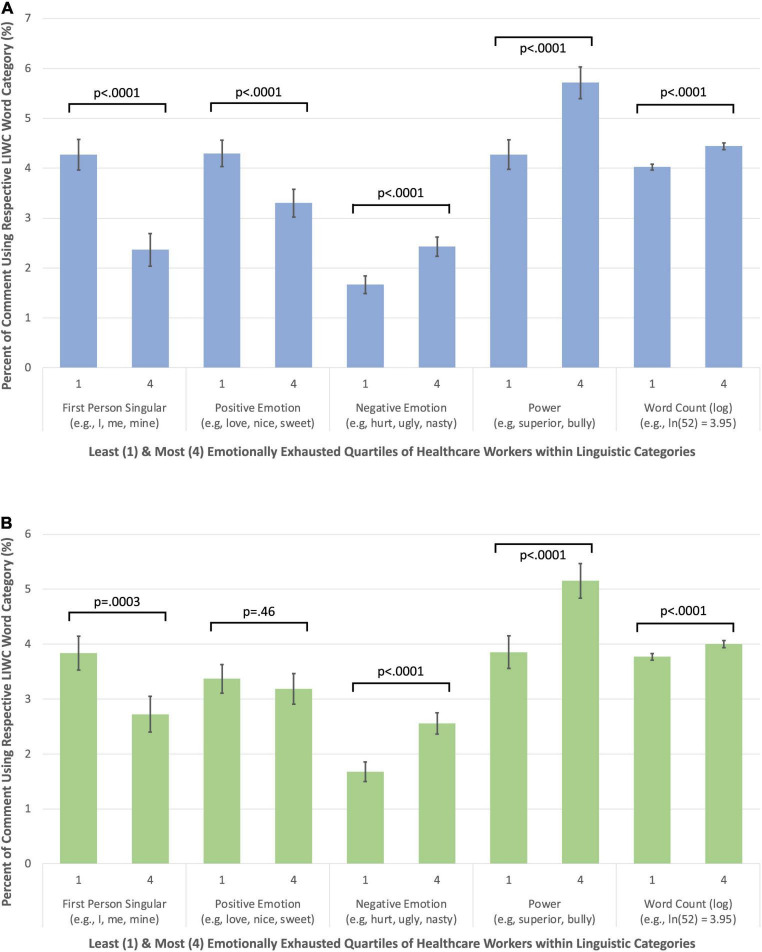
**(A)** The linguistic profiles of the least and most emotionally exhausted healthcare workers (HCWs) [2019 emotional exhaustion (EE) and 2019 comments]. **(B)** The linguistic profiles of the least and most emotionally exhausted HCWs (2020 EE and 2020 comments).

Same-year, pairwise associations of the writing of the lowest and highest EE quartiles controlling for Word Count (Table 2) revealed emotionally exhausted HCWs used more words overall (*p* < 0.001), Negative Emotion (*p* < 0.001), and references to Power (*p* < 0.001). The highest EE quartile also used less First Person Singular (*p* < 0.001) and Positive Emotion (*p*_2019_ < 0.001, *p*_2020_ = 0.46). These effects were consistent for 2019 and 2020 data, as highlighted in Figure 1. There were no significant differences between lowest and highest EE quartiles for First Person Plural, Present Focus, Past Focus, and Social words. Analyses controlling for age, sex, and race revealed the same pattern of results (Supplementary Appendix Table 2).

**TABLE 2:** Pair-wise associations between the frequencies of nine linguistic categories in healthcare worker (HCW) comments across levels of emotional exhaustion (EE).

Linguistic category (example words)	Quartile	Same-year comparisons				Across-year comparisons			
			
	1: min EE	2019 comments and 2019 emotional exhaustion		2020 comments and 2020 emotional exhaustion		2019 comments predicting 2020 emotional exhaustion		2019 emotional exhaustion predicting 2020 comments	
					
	4: max EE	Percent (95% CI)	*p*	Percent (95% CI)	*p*	Percent (95% CI)	*p*	Percent (95% CI)	*p*
Word count (log)*	1	**4.0% (4.0, 4.1)**	**<0.0001**	**3.8% (3.7, 3.8)**	**<0.0001**	**4.0% (3.9, 4.1)**	**<0.0001**	3.9% (3.8, 3.9)	0.10
[e.g., ln(52) = 3.95]	4	**4.4% (4.4, 4.5)**		**4.0% (3.9, 4.1)**		**4.5% (4.4, 4.6)**		4.0% (3.9, 4.1)	
First person singular	1	**4.3% (4.0, 4.6)**	**<0.0001**	**3.8% (3.4, 4.3)**	**0.0003**	**4.2% (3.7, 4.7)**	**<0.0001**	3.6% (3.0, 4.2)	0.12
(e.g., I, me, mine)	4	**2.4% (2.0, 2.7)**		**2.7% (2.3, 3.2)**		**2.6% (2.1, 3.1)**		2.9% (2.3, 3.5)	
First person plural	1	2.5% (2.2, 2.7)	0.42	2.3% (1.9, 2.6)	0.04	2.3% (2.0, 2.7)	0.12	2.3% (1.9, 2.8)	0.64
(e.g., we, us, our)	4	2.6% (2.3, 2.9)		2.8% (2.4, 3.1)		2.8% (2.4, 3.2)		2.5% (2.0, 2.9)	
Present focus (log)	1	2.6% (2.6, 2.6)	0.02	2.5% (2.5, 2.6)	0.004	2.6% (2.5, 2.6)	0.72	2.6% (2.5, 2.6)	0.20
(e.g., today, is, now)	4	2.6% (2.6, 2.7)		2.6% (2.6, 2.7)		2.6% (2.5, 2.7)		2.6% (2.6, 2.7)	
Past focus	1	2.2% (2.0, 2.4)	0.63	2.8% (2.5, 3.1)	0.03	2.3% (2.0, 2.6)	0.11	2.8% (2.4, 3.2)	0.40
(e.g., ago, did, talked)	4	2.1% (1.9, 2.3)		2.3% (2.0, 2.7)		1.9% (1.6, 2.2)		2.5% (2.1, 3.0)	
Positive emotion	1	**4.3% (4.0, 4.6)**	**<0.0001**	3.4% (3.0, 3.7)	0.46	4.2% (3.8, 4.6)	0.07	3.2% (2.7, 3.6)	0.27
(e.g., love, nice, sweet)	4	**3.3% (3.0, 3.6)**		3.2% (2.8, 3.5)		3.6% (3.2, 4.1)		2.8% (2.3, 3.3)	
Negative Emotion	1	**1.7% (1.5, 1.8)**	**<0.0001**	**1.7% (1.4, 1.9)**	**<0.0001**	**1.4% (1.2, 1.7)**	**<0.0001**	2.0% (1.6, 2.3)	0.09
(e.g., hurt, ugly, nasty)	4	**2.4% (2.2, 2.6)**		**2.6% (2.3, 2.8)**		**2.4% (2.1, 2.7)**		2.4% (2.1, 2.8)	
Social	1	8.8% (8.4, 9.2)	0.69	7.4% (6.8, 7.9)	0.03	8.6% (8.0, 9.2)	0.91	7.4% (6.6, 8.1)	0.32
(e.g., mate, talk, they)	4	8.6% (8.2, 9.1)		8.3% (7.7, 8.8)		8.7% (8.0, 9.3)		7.9% (7.1, 8.7)	
Power	1	**4.3% (4.0, 4.6)**	**<0.0001**	**3.9% (3.4, 4.3)**	**<0.0001**	**4.3% (3.9, 4.8)**	**0.0002**	4.4% (3.8, 5.0)	0.05
(e.g., own, order, allow)	4	**5.7% (5.4, 6.0)**		**5.2% (4.7, 5.6)**		**5.6% (5.1, 6.1)**		5.2% (4.6, 5.8)	

*All categories, except Word count, controlled for Word count. Associations meeting the Bonferroni threshold for significance *p* < 0.0014 are bolded.

#### 3.4.2 Across year associations

**FIGURE 2:**
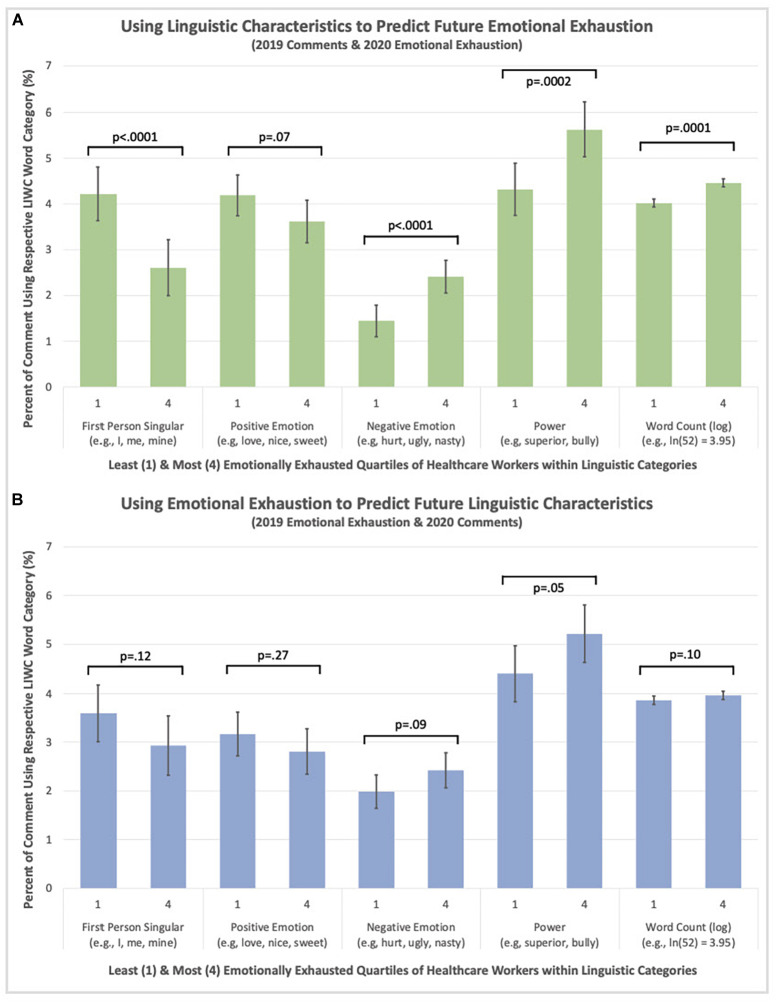
**(A)** Using linguistic characteristics to predict future EE (2019 comments and 2020 EE). **(B)** Using emotional exhaustion (EE) to predict future linguistic characteristics (2019 EE and 2020 comments).

Pairwise associations of the lowest and highest EE quartiles (Table 2) controlling for Word Count revealed emotionally exhausted HCWs in 2020 had used more words (*p* < 0.001), Negative Emotions (*p* < 0.001), and references to Power (*p* < 0.001) in prior (2019) comments. The highest EE quartile also used less First Person Singular words (*p* < 0.001). Figure 2 highlights these trends. First Person Plural, Present Focus, Past Focus, Positive Emotion, and Social words did not significantly differ between quartiles. Conversely, none of the 2020 linguistic categories significantly differed by 2019 EE quartiles. See Supplementary Appendix Figures 1, 2 for side-by-side visualizations of within and across year data. Analyses controlling for age, sex, and race revealed the same pattern of results (Supplementary Appendix Table 2).

#### 3.4.3 Example statements for significant linguistic categories

Statements that used high vs. low frequencies of the five significant linguistic categories demonstrated notable differences in tone and content. Table 3 provides example statements of the highest and lowest proportions of Positive Emotion, Negative Emotion, Power, and First Person Singular.

**TABLE 3:** Representative comments from the frequent and infrequent use of the linguistic categories.

**Frequent use of positive emotion**
I love my job. I find satisfaction in my work and assisting patient’s in getting better. My colleagues/co-workers work well together.
I love my job. No work environment is perfect, because people aren’t perfect, but I have a pretty great setting that I am grateful for.
**Infrequent use of positive emotion**
The “Felt frustrated by technology” is interpreted in this case in the context of frustration with old, damaged, or bad software/hardware technology.
Main concern is that management allows particular favorites to abuse everyday work policies without consequences. Especially while they are some of the high paid employees of the office.
**Frequent use of negative emotion**
Inadequate staffing, equipment, and wait times between report and delivery of new Pts all contribute to the stress level, Pt complaints, and overall unsafe feelings and practices in this facility.
I wish we could have employee coverage when a co-worker calls in sick instead of having to work short staffed. Employee’s and Patient’s are the one’s suffering and frustrated and We are NOT doing What’s Best, it makes — Corporation look BAD!
**Infrequent use of negative emotion**
As a team our office works very well together. we compliment each others work style and are always looking for ways to improve ourselves and the patient experience.
It would be much better if we could communicate better with our manager. Our manager is off site and our only means of communication are mainly by emails or phone and responses are slow.
**Frequent use of power**
I feel my direct managers are trying to help turn our culture in our department, but are unable to d/t upper management constraints.
Staffing issues are a constant problem. WE consider this a huge safety issue. Management does not. What employees consider important for patients and themselves is not a high priority with management.
**Infrequent use of power**
We come to this building in the dark and leave in the dark, there isn’t any security at this building. There are —% women in this building and we are in an open space where people walk through our parking lot all the time.
I feel very lucky to be working here doing the job that I am doing. I love my job. What could be better is if people were held accountable for the quality of their work.
**Frequent use of first person singular**
I changed positions and it is a perfect fit for me. I love what I do and have a fantastic leader and coworkers.
I love my job. I find satisfaction in my work and assisting patient’s in getting better. My colleagues/co-workers work well together.
**Infrequent use of first person singular**
The senior management of the hospital has been trying to cut budgets and save money for such a period of time, it has hurt patient safety and safe staffing practices.
Poor or inadequate staff is a major safety concern. Not only for our patients but also for the nurses mental health. Nurses are getting burnt out with how poorly staffed and busy we are becoming.

## 4 Discussion

The current study examined associations between linguistic categories used in HCW writings, and their past, current, and future EE scores. Using a large, 2-year dataset, we found Word Count, First Person Singular, Power, and Positive/Negative Emotions were consistently associated with EE–with and without controlling for age, gender, race, and WC. These linguistic categories were mostly consistent in predicting current and future EE (a year later). However, EE did not predict future use of linguistic categories. In other words, language predicted subsequent EE, but EE did not predict subsequent language use. To our knowledge, this is the first study to show that the language used by HCWs can predict subsequent well-being. If these findings are replicated, they raise questions about the etiology of EE and the potential for linguistic analyses to help analyze well-being and inform targeted, preventative interventions.

Overall, linguistic categories mostly linked to EE in the hypothesized direction. In line with prior research on mental health and work environments, the HCWs experiencing *high* EE used more words and negative emotion, and less positive emotions (21, 23, 31, 43). Depression and EE appear to share this linguistic pattern, potentially reflecting their overlap of emotional suffering (17, 44, 45).

Contrary to our hypothesis, more references to oneself (i.e., First Person Singular) were associated with *lower* EE, both within the same year and the following year. This differs from depression, where greater use of first person can signal self-focused rumination and social detachment (25, 43, 46–48). The study’s sample comments suggest First Person Singular could indicate increased perceptions of autonomy, competency, and positive relationships with colleagues, factors which might lower EE. For example, “I enjoy my job. I am fortunate to have two managers and my supervisors that I report to. They address my concerns and I feel they always listen to me and help me find answers to my questions…” “I like my job and the people I interact with while doing my job. It is a hard and busy job but I do not mind makes time go buy fast and keeps me focused I think I adapt well to whatever comes my way.” Further examples are available in Table 3. These findings parallel Self-Determination Theory (SDT), which posits people who experience autonomy, competence, and relatedness have lower burnout (49). Alternatively, HCWs may have simply been primed by the first-person focus of SCORE’s individual items (e.g., “I feel burned out from my job”). Moreover, the comment prompt (“Do you have any other comments, questions, or concerns?”) may have elicited a first-person framework unlike natural language or conversation, as prior studies have shown the focus of writing can influence linguistic choices (31, 50).

Words referencing power (e.g., own, order, and allow) predicted *higher* EE. Increased references to power may highlight how excess job demands and workload without compensating supporting resources may lead to EE (51–53). For example, “Under staffing is an extremely large issue and could help with the majority of the problems working at this facility.” Moreover, since attention has been shown to follow power, the references may highlight subordinates paying attention to the way their leaders (negatively) influence their day to day experience (33). For example, “Don’t feel the same amount of respect/support/transparency from executive leadership as in years past.”

Four hypothesized linguistic categories (Present Focus, Past Focus, First Person Plural, and Social) did not associate significantly with EE. We expected Present Focus to reflect the positive influence of mindfulness and being present; we expected Past Focus to reflect brooding on the past, which has been shown to associate with a greater likelihood for depression (46–48).

We expected First Person Plural and Social words to capture the protective effects of a team-mindset and supportive relationships on EE. However, these effects may have been obscured by the inability of the linguistic software to distinguish between the positive presence of relationships (e.g., “we are a great team”) or the opposite (e.g., “we are constantly understaffed”). A thematic analysis that evaluates the positivity/negativity of comments using First Person Plural and Social words might uncover a more nuanced relationship with EE.

A noteworthy element of these results was that linguistic categories predicted future EE, but EE did not predict subsequent linguistic categories. This unidirectional link whereby language predicts future exhaustion suggests that cognitions (conscious and/or unconscious) influence the use of language and precede the development of EE. If true, this link indicates that people at risk of developing EE could be identified early based on their linguistic patterns. This early warning system could potentially prevent further suffering through early detection and treatment. To identify vulnerable HCWs and work settings, and to act more prophylactically with respect to well-being resources would be a remarkable advance in current efforts to support the workforce. A lofty aspiration would be to use a language of EE to identify individuals and work settings at risk for increasing EE, spurring a host of targeted individual, work-setting, and organizational resources and interventions (23, 54–63).

To date, only two studies have investigated associations between linguistic categories and HCW EE (23, 24). This study adds a third piece of preliminary evidence, using a 3–5 times larger dataset that features a greater diversity of HCW roles. Individual HCW responses were linked across time, enabling the first investigation of how linguistic categories of HCW comments associate with prior and future EE. The study’s large sample size, longitudinal nature, conservative statistical analysis, and consistency of associations between linguistic categories and current, prior, and future EE lend confidence to the findings.

### 4.1 Limitations

This study provides preliminary evidence for linguistic markers of EE, using a unique multi-year sample. Despite this ability in this dataset to assess HCW language and EE over time, this study has limitations including selection bias, restrictions in range, limits to generalizability, and reductions in statistical power.

Due to survey management choices in this particular health system, physicians did not participate. As a voluntary self-report survey, these data are subject to potential selection and desirability response biases. Prior research has found linguistic categories are relatively stable, but can vary across the context of language use (31, 40). Thus, the comment prompt at the end of a safety culture and well-being survey may have influenced the HCWs use of language. This health system was in the most emotionally exhausted quartile relative to other US healthcare facilities in Safe and Reliable Healthcare’s benchmarking database, restricting our data analysis to a higher EE subset. Though necessary to establish adequate reliability for the linguistic analyses, excluding comments with a WC < 20 yielded smaller samples with even higher EE. Although breaking EE into quartiles is a methodologically acceptable approach, it further reduced the power of the study. Overall, the reductions in power and restricted EE range likely reduced our ability to find associations between linguistic categories and EE.

Future studies should include physicians, use larger samples of HCW comments, and target institutions and work settings with more typical EE levels to avoid restriction of range issues. However, even with a restricted range and reduced power, we were able to identify clear associations between language categories and EE. Additional investigations across different prompts, demographics, and settings could clarify the reliability of linguistic categories associating with EE. Combining quantitative and qualitative text analyses might yield further insight into the psychological processes and environmental influences contributing to EE. Given further research on this topic, linguistic categories could 1 day serve as markers for identifying current and future EE. Analyzing linguistic categories from consensually collected writing samples (e.g., charts, emails) to signal EE risk could help mitigate survey fatigue from repeatedly assessing burnout *via* scales (35).

## 5 Conclusion

This study investigated associations between linguistic categories in HCW writing samples and their EE within and across 2 years. Comments using more words, Negative Emotion, and references to Power, and less Positive Emotion and First Person Singular predicted higher current and future EE. Unidirectionally, language predicted current EE, but EE did not predict subsequent language. These results help to establish a line of research into the language of HCW EE, with the potential to offer new insights into causes, consequences, interventions, and metrics of HCW well-being.

## Data availability statement

The data analyzed in this study is subject to the following licenses/restrictions: Upon reasonable requests, the data can be made available by the corresponding author. Requests to access these datasets should be directed to JS, bryan.sexton@duke.edu.

## Ethics statement

The studies involving human participants were reviewed and approved by the Institutional Review Board at Duke University Medical Center (Pro00083427). Written informed consent for participation was not required for this study in accordance with the national legislation and the institutional requirements.

## Author contributions

FFB, KCA, and JBS: study conception, design, analysis, and interpretation of results. JP and ASF: data collection. All authors drafted the manuscript preparation, reviewed the results, and approved the final version of the manuscript.

## References

[1] Wurm W Vogel K Holl A Ebner C Bayer D Mörkl S Depression-burnout overlap in physicians. *PLoS One.* (2016). 11:e0149913. 10.1111/ajad.12173 26930395 PMC4773131

[2] Oreskovich M Shanafelt T Dyrbye L Tan L Sotile W Satele D The prevalence of substance use disorders in American physicians. *Am J Addict.* (2015) 24:30–8. 10.1080/13811110802325349 25823633

[3] van der Heijden F Dillingh G Bakker A Prins J. Suicidal thoughts among medical residents with burnout. *Arch Suicide Res.* (2008) 12:344–6. 10.1097/ACM.0b013e31819391bb 18828037

[4] Goebert D Thompson D Takeshita J Beach C Bryson P Ephgrave K Depressive symptoms in medical students and residents: a multischool study. *Acad Med.* (2009) 84:236–41. 10.1177/0013164410391579 19174678

[5] Wheeler D Vassar M Worley J Barnes LLB. A reliability generalization meta-analysis of coefficient alpha for the maslach burnout inventory. *Educ Psychol Meas.* (2011) 71:231–44. 10.1007/s11606-016-3856-2 27612486 PMC5215160

[6] Linzer M Poplau S Brown R Grossman E Varkey A Yale S Do work condition interventions affect quality and errors in primary care? Results from the healthy work place study. *J Gen Intern Med.* (2017) 32:56–61. 10.1371/journal.pone.0159015 27612486 PMC5215160

[7] Hall L Johnson J Watt I Tsipa A O’Connor D. Healthcare staff wellbeing, burnout, and patient safety: a systematic review. *PLoS One.* (2016) 11:e0159015. 10.7326/M19-1152 27391946 PMC4938539

[8] Tawfik D Scheid A Profit J Shanafelt T Trockel M Adair K Evidence relating healthcare provider burnout and quality of care: a systematic review and meta-analysis. *Ann Intern Med.* (2019) 171:555–67. 10.1007/s11606-016-3886-9 31590181 PMC7138707

[9] Salyers M Bonfils K Luther L Firmin R White D Adams E The relationship between professional burnout and quality and safety in healthcare: a meta-analysis. *J Gen Intern Med.* (2017) 32:475–82. 10.1016/j.ajic.2012.02.029 27785668 PMC5377877

[10] Cimiotti J Aiken L Sloane D Wu E. Nurse staffing, burnout, and health care–associated infection. *Am J Infect Control.* (2012) 40:486–90. 10.1097/SLA.0b013e3181bfdab3 22854376 PMC3509207

[11] Shanafelt T Balch C Bechamps G Russell T Dyrbye L Satele D Burnout and medical errors among American surgeons. *Ann Surg.* (2010) 251:995–1000. 10.1111/jonm.12589 19934755

[12] Zhang Y Han W Qin W Yin H Zhang C Kong C Extent of compassion satisfaction, compassion fatigue and burnout in nursing: a meta-analysis. *J Nurs Manag.* (2018) 26:810–9. 10.1001/jama.2018.12777 30129106

[13] Rotenstein L Torre M Ramos M Rosales R Guille C Sen S Prevalence of burnout among physicians. *JAMA.* (2018) 320:1131–50. 10.1136/bmj.e1717 30326495 PMC6233645

[14] Aiken LH Sermeus W Van den Heede K Sloane D Busse R McKee M Patient safety, satisfaction, and quality of hospital care: cross sectional surveys of nurses and patients in 12 countries in Europe and the United States. *BMJ.* (2012) 344:e1717. 10.1001/jamanetworkopen.2022.32748 22434089 PMC3308724

[15] Sexton J Adair K Proulx J Profit J Cui X Bae J Emotional exhaustion among US health care workers before and during the COVID-19 pandemic, 2019-2021. *JAMA Netw Open.* (2022) 5:e2232748. 10.1016/j.genhosppsych.2020.06.007 36129705 PMC9494188

[16] Shechter A Diaz F Moise N Anstey D Ye S Agarwal S Psychological distress, coping behaviors, and preferences for support among New York healthcare workers during the COVID-19 pandemic. *Gen Hosp Psychiatry.* (2020) 66:1–8. 10.3389/fpsyg.2020.01684 32590254 PMC7297159

[17] Giusti E Pedroli E D’Aniello G Stramba Badiale C Pietrabissa G Manna C The psychological impact of the COVID-19 outbreak on health professionals: a cross-sectional study. *Front Psychol.* (2020) 11:1684. 10.1097/JHM-D-18-00209 32754102 PMC7366071

[18] Shanafelt T Swensen S Woody J Levin J Lillie J. Physician and nurse well-being: seven things hospital boards should know. *J Healthc Manag.* (2018) 63:363–9. 10.1016/j.mayocp.2016.10.004 30418362

[19] Shanafelt T Noseworthy J. Executive leadership and physician well-being. *Mayo Clin Proc.* (2017) 92:129–46. 10.1136/bmjopen-2022-065320 27871627

[20] Ellis L Pomare C Churruca K Carrigan A Meulenbroeks I Saba M Predictors of response rates of safety culture questionnaires in healthcare: a systematic review and analysis. *BMJ Open.* (2022) 12:e065320. 10.1186/s12913-018-3743-0 36113948 PMC9486325

[21] Adair K Quow K Frankel A Mosca P Profit J Hadley A The improvement readiness scale of the SCORE survey: a metric to assess capacity for quality improvement in healthcare. *BMC Health Serv Res.* (2018) 18:975. 10.1177/0261927X09351676 PMC6296100 30558593

[22] Tausczik Y Pennebaker J. The psychological meaning of words: LIWC and computerized text analysis methods. *J Lang Soc Psychol.* (2010) 29:24–54. 10.2196/15562 32406864 PMC7256751

[23] Adair K Rodriguez-Homs L Masoud S Mosca P Sexton J. Gratitude at work: prospective cohort study of a web-based, single-exposure well-being intervention for health care workers. *J Med Internet Res.* (2020) 22:e15562. 10.1177/1359105317727841 32406864 PMC7256751

[24] Gandino G Di Fini G Bernaudo A Paltrinieri M Castiglioni M Veglia F. The impact of perinatal loss in maternity units: a psycholinguistic analysis of health professionals’ reactions. *J Health Psychol.* (2020) 25:640–51. 10.1073/pnas.1802331115 28854811

[25] Eichstaedt J Smith R Merchant R Ungar L Crutchley P Preoţiuc-Pietro D Facebook language predicts depression in medical records. *Proc Natl Acad Sci USA.* (2018) 115:11203–8. 10.3115/v1/W14-3214 30322910 PMC6217418

[26] Schwartz H Eichstaedt J Kern M Park G Sap M Stillwell D Towards assessing changes in degree of depression through Facebook. In: *Proceedings of the Workshop on Computational Linguistics and Clinical Psychology: From Linguistic Signal to Clinical Reality.* Baltimore, MD: Association for Computational Linguistics (2014). p. 118–25. 10.1038/s41598-017-12961-9

[27] Reece A Reagan A Lix K Dodds P Danforth C Langer E. Forecasting the onset and course of mental illness with Twitter data. *Sci Rep.* (2017) 7:13006. 10.1007/s10459-017-9775-0 29021528 PMC5636873

[28] Dennis A Foy M Monrouxe L Rees C. Exploring trainer and trainee emotional talk in narratives about workplace-based feedback processes. *Adv Health Sci Educ.* (2018) 23:75–93. 10.2196/jmir.5042 28456856 PMC5801389

[29] Xu R Zhang Q. Understanding online health groups for depression: social network and linguistic perspectives. *J Med Internet Res.* (2016) 18:e63. 10.1609/icwsm.v2i1.18623 PMC4807247 26966078

[30] Ramirez-Esparza N Chung C Kacewic E Pennebaker J. The psychology of word use in depression forums in English and in Spanish: testing two text analytic approaches. *Proc Int AAAI Conf Web Soc Media.* (2021) 2:102–8. 10.1371/journal.pone.0207814 30475918 PMC6258120

[31] Himmelstein P Barb S Finlayson M Young K. Linguistic analysis of the autobiographical memories of individuals with major depressive disorder. *PLoS One.* (2018) 13:e0207814. 10.1080/14719037.2021.1900351 PMC6258120 30475918

[32] Battaglio R Belle N Cantarelli P. Self-determination theory goes public: experimental evidence on the causal relationship between psychological needs and job satisfaction. *Public Manag Rev.* (2022) 24:1411–28. 10.1037/0003-066X.48.6.621 8328729

[33] Fiske S. Controlling other people: the impact of power on stereotyping. *Am Psychol.* (1993) 48:621–8. 8328729 10.1037//0003-066x.48.6.621

[34] Sexton J Frankel A Leonard M Adair K. *SCORE: Assessment of your work setting Safety, Communication, Operational Reliability, and Engagement. Report No.: 19–5.* Durham, NC: Duke Center for Healthcare Safety and Quality (2019). 21 p.

[35] Maslach C Jackson S Leiter M. Maslach burnout inventory: third edition. In: Zalaquett C Wood RJ editors. *Evaluating Stress: A Book of Resources.* Lanham, MD: Scarecrow Education (1997). p. 191–218. 10.1016/S1553-7250(13)39028-X

[36] Block M Ehrenworth J Cuce V Ng’ang’a N Weinbach J Saber S Measuring handoff quality in labor and delivery: development, validation, and application of the coordination of handoff effectiveness questionnaire (CHEQ). *Jt Comm J Qual Patient Saf.* (2013) 39:213–20. 10.1136/bmjqs-2014-002831 23745480

[37] Profit J Sharek P Amspoker A Kowalkowski M Nisbet C Thomas E Burnout in the NICU setting and its relation to safety culture. *BMJ Qual Saf.* (2014) 23:806–13. 10.1136/bmjqs-2016-006399 24742780 PMC4167972

[38] Sexton J Adair K Leonard M Frankel T Proulx J Watson S Providing feedback following leadership walkrounds is associated with better patient safety culture, higher employee engagement and lower burnout. *BMJ Qual Saf.* (2018) 27:261–70. 10.1136/bmjqs-2016-006399 PMC5867443 28993441

[39] Pennebaker J Booth R Boyd R Francis M. *Linguistic Inquiry and Word Count: LIWC2015.* Austin, TX: Pennebaker Conglomerates (2021).

[40] Pennebaker J Boyd R Jordan K Blackburn K. *The Development and Psychometric Properties of LIWC2015.* Austin TX: The University of Texas at Austin (2015).

[41] Pennebaker Conglomerates Inc. *LIWC - How it Works.* Austin, TX: Pennebaker Conglomerates Inc (2019). 10.1075/scl.85

[42] Whitt R. *Diachronic Corpora, Genre, and Language Change.* Amsterdam: John Benjamins Publishing Company (2018). 347 p. 10.1080/02699930441000030

[43] Rude S Gortner E Pennebaker J. Language use of depressed and depression-vulnerable college students. *Cogn Emot.* (2004) 18:1121–33. 10.13075/ijomeh.1896.01323 30855601

[44] Golonka K Mojsa-Kaja J Blukacz M Gawłowska M Marek T. Occupational burnout and its overlapping effect with depression and anxiety. *Int J Occup Med Environ Health.* (2019) 32:229–44. 10.1016/j.cpr.2015.01.004 30855601

[45] Bianchi R Schonfeld I Laurent E. Burnout–depression overlap: a review. *Clin Psychol Rev.* (2015) 36:28–41. 10.1037/0022-3514.69.1.176 25638755

[46] Lyubomirsky S Nolen-Hoeksema S. Effects of self-focused rumination on negative thinking and interpersonal problem solving. *J Pers Soc Psychol.* (1995) 69:176–90. 10.3389/fpsyg.2012.00020 7643299

[47] Sorg S Vögele C Furka N Meyer A. Perseverative thinking in depression and anxiety. *Front Psychol.* (2012) 3:20. 10.1037/0021-843X.117.2.314 22347869 PMC3277932

[48] Moberly N Watkins E. Ruminative self-focus and negative affect: an experience sampling study. *J Abnorm Psychol.* (2008) 117:314–23. 10.1080/1359432X.2011.632161 18489207 PMC2672047

[49] Fernet C Austin S Trépanier S Dussault M. How do job characteristics contribute to burnout? Exploring the distinct mediating roles of perceived autonomy, competence, and relatedness. *Eur J Work Organ Psychol.* (2013) 22:123–37. 10.2307/20445398

[50] Kahn J Tobin R Massey A Anderson J. Measuring emotional expression with the linguistic inquiry and word count. *Am J Psychol.* (2007) 120:263–86. 10.1186/s12889-017-4153-7 17650921

[51] Aronsson G Theorell T Grape T Hammarström A Hogstedt C Marteinsdottir I A systematic review including meta-analysis of work environment and burnout symptoms. *BMC Public Health.* (2017) 17:264. 10.1177/0046958017724944 28302088 PMC5356239

[52] Mudallal R Othman W Al Hassan N. Nurses’ burnout: the influence of leader empowering behaviors, work conditions, and demographic traits. *Inquiry.* (2017) 54:6958017724944. 10.1007/s00420-021-01669-z 28844166 PMC5798741

[53] Barello S Caruso R Palamenghi L Nania T Dellafiore F Bonetti L Factors associated with emotional exhaustion in healthcare professionals involved in the COVID-19 pandemic: an application of the job demands-resources model. *Int Arch Occup Environ Health.* (2021) 94:1751–61. 10.1007/s40596-017-0868-0 33660030 PMC7928172

[54] Pospos S Young I Downs N Iglewicz A Depp C Chen J Web-based tools and mobile applications to mitigate burnout, depression, and suicidality among healthcare students and professionals: a systematic review. *Acad Psychiatry.* (2018) 42:109–20. 10.1097/DCR.0000000000000844 29256033 PMC5796838

[55] Rothenberger D. Physician burnout and well-being: a systematic review and framework for action. *Dis Colon Rectum.* (2017) 60:567–76. 10.1001/jamanetworkopen.2020.9385 28481850

[56] West C Dyrbye L Sinsky C Trockel M Tutty M Nedelec L Resilience and burnout among physicians and the general US working population. *JAMA Netw Open.* (2020) 3:e209385. 10.1002/14651858.CD013779 32614425 PMC7333021

[57] Pollock A Campbell P Cheyne J Cowie J Davis B McCallum J Interventions to support the resilience and mental health of frontline health and social care professionals during and after a disease outbreak, epidemic or pandemic: a mixed methods systematic review. *Cochrane Database Syst Rev.* (2020) 11:CD013779. 10.1097/MD.0000000000020992 33150970 PMC8226433

[58] Zhang X Song Y Jiang T Ding N Shi T. Interventions to reduce burnout of physicians and nurses: an overview of systematic reviews and meta-analyses. *Medicine.* (2020) 99:e20992. 10.1038/s41372-021-01100-y 32590814 PMC7328917

[59] Profit J Adair K Cui X Mitchell B Brandon D Tawfik D Randomized controlled trial of the “WISER” intervention to reduce healthcare worker burnout. *J Perinatol.* (2021) 41:2225–34. 10.1136/bmjopen-2018-022695 34366432 PMC8440181

[60] Sexton J Adair K. Forty-five good things: a prospective pilot study of the three good things well-being intervention in the USA for healthcare worker emotional exhaustion, depression, work–life balance and happiness. *BMJ Open.* (2019) 9:e022695. 10.1080/17439760.2020.1789707 30898795 PMC6475256

[61] Adair K Kennedy L Sexton J. Three good tools: positively reflecting backwards and forwards is associated with robust improvements in well-being across three distinct interventions. *J Posit Psychol.* (2020) 15:613–22. 10.1097/PTS.0000000000001048 34295357 PMC8294345

[62] Adair K Heath A Frye M Frankel A Proulx J Rehder K The psychological safety scale of the safety, communication, operational, reliability, and engagement (SCORE) survey: a brief, diagnostic, and actionable metric for the ability to speak up in healthcare settings. *J Patient Saf.* (2022) 18:513–20. 35985041 10.1097/PTS.0000000000001048 PMC9422763

[63] Sexton JB Adair KC Cui X Tawfik DS Profit J. Effectiveness of a bite-sized web-based intervention to improve healthcare worker wellbeing: a randomized clinical trial of WISER. *Front Public Health*. (2020) 10:1016407. 10.3389/fpubh.2022.1016407 PMC9773843 36568789

