# Corrigendum: The language of healthcare worker emotional exhaustion: a linguistic analysis of longitudinal survey

**DOI:** 10.3389/fpsyt.2023.1243602

**Published:** 2023-08-03

**Authors:** Franz F. Belz, Kathryn C. Adair, Joshua Proulx, Allan S. Frankel, J. Bryan Sexton

**Affiliations:** ^1^Duke School of Medicine, Duke University, Durham, NC, United States; ^2^Duke Center for Healthcare Safety and Quality, Duke University Health System, Durham, NC, United States; ^3^Safe and Reliable Healthcare, Evergreen, CO, United States

**Keywords:** burnout, emotional exhaustion, stress, well-being, LIWC, linguistic analyses, healthcare worker (HCW), healthcare quality

In the published article, there was an error in the legend for Figure 2 as published. The labels for Figure 2 are flipped and thus incorrectly communicate key findings from the paper. The corrected legend appears below.

**Figure 2:**
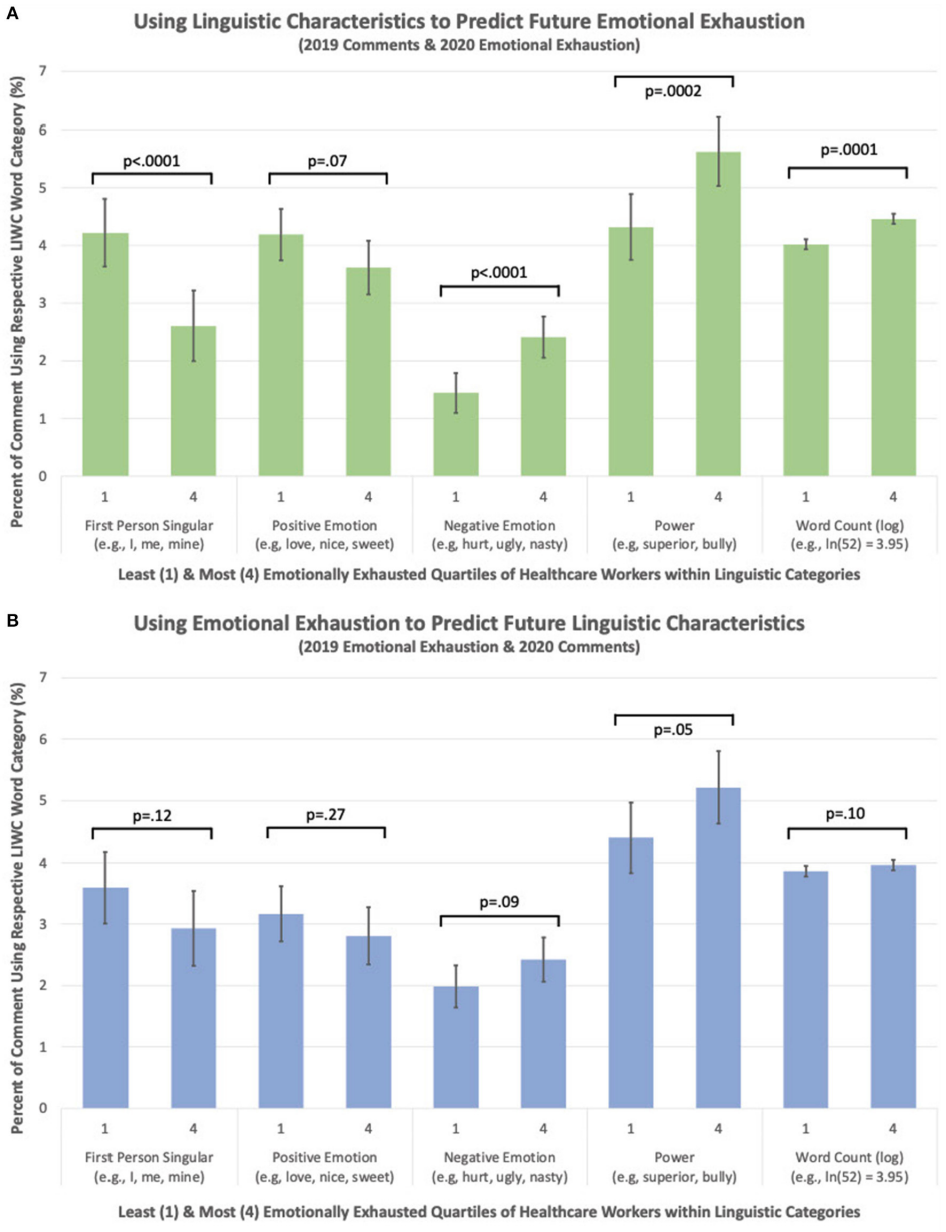
**(A)** Using linguistic characteristics to predict future EE (2019 comments and 2020 EE). **(B)** Using emotional exhaustion (EE) to predict future linguistic characteristics (2019 EE and 2020 comments).

In the published article, there was an error in Table 3 as published. “Frequent use of positive emotion” is incorrectly formatted as a title, rather than the table section label it should be. The corrected Table 3 and its caption “Representative comments from the frequent and infrequent use of the linguistic categories” appear below.

**Table 3:** Representative comments from the frequent and infrequent use of the linguistic categories.

**Frequent use of positive emotion**
I love my job. I find satisfaction in my work and assisting patient's in getting better. My colleagues/co-workers work well together. • I love my job. No work environment is perfect, because people aren't perfect, but I have a pretty great setting that I am grateful for.
**Infrequent use of positive emotion**
The “Felt frustrated by technology” is interpreted in this case in the context of frustration with old, damaged, or bad software/hardware technology. • Main concern is that management allows particular favorites to abuse everyday work policies without consequences. Especially while they are some of the high paid employees of the office.
**Frequent use of negative emotion**
Inadequate staffing, equipment, and wait times between report and delivery of new Pts all contribute to the stress level, Pt complaints, and overall unsafe feelings and practices in this facility. • I wish we could have employee coverage when a co-worker calls in sick instead of having to work short staffed. Employee's and Patient's are the one's suffering and frustrated and We are NOT doing What's Best, it makes—Corporation look BAD!
**Infrequent use of negative emotion**
As a team our office works very well together. we compliment each others work style and are always looking for ways to improve ourselves and the patient experience. • It would be much better if we could communicate better with our manager. Our manager is off site and our only means of communication are mainly by emails or phone and responses are slow.
**Frequent use of power**
I feel my direct managers are trying to help turn our culture in our department, but are unable to d/t upper management constraints. • Staffing issues are a constant problem. WE consider this a huge safety issue. Management does not. What employees consider important for patients and themselves is not a high priority with management.
**Infrequent use of power**
We come to this building in the dark and leave in the dark, there isn't any security at this building. There are —% women in this building and we are in an open space where people walk through our parking lot all the time. • I feel very lucky to be working here doing the job that I am doing. I love my job. What could be better is if people were held accountable for the quality of their work.
**Frequent use of first person singular**
I changed positions and it is a perfect fit for me. I love what I do and have a fantastic leader and coworkers. • I love my job. I find satisfaction in my work and assisting patient's in getting better. My colleagues/co-workers work well together.
**Infrequent use of first person singular**
The senior management of the hospital has been trying to cut budgets and save money for such a period of time, it has hurt patient safety and safe staffing practices. • Poor or inadequate staff is a major safety concern. Not only for our patients but also for the nurses mental health. Nurses are getting burnt out with how poorly staffed and busy we are becoming.

The authors apologize for this error and state that this does not change the scientific conclusions of the article in any way. The original article has been updated.

