# Supplementary material for: The language of healthcare worker emotional exhaustion: A linguistic analysis of longitudinal survey
Source: Front Psychiatry. 2022 Dec 16;13:1044378. doi: 10.3389/fpsyt.2022.1044378 (PMC9800594; doi:10.3389/fpsyt.2022.1044378)
Supplement: Supplementary file 1 [file Data_Sheet_1.pdf]

App. Table 1. Frequencies of All Linguistic Categories in Healthcare Worker Comments Across Levels of Emotional Exhaustion (EE) Controlling for Word Count Only

Linguistic Category (example words)	Quartile 1: min EE 4: max EE	Same-Year Comparisons				Across-Year Comparisons			
		**2019 Comments & 2019 EE**		**2020 Comments & 2020 EE**		**2019 Comments & 2020 EE**		**2019 EE & 2020 Comments**	
		Percent (95% CI)	p	Percent (95% CI)	p	Percent (95% CI)	p	Percent (95% CI)	p
**Word Count (log)**	1	**4.0% (4.0,4.1)**	**<.0001**	**3.8% (3.7,3.8)**	**<.0001**	**4.0% (3.9,4.1)**	**<.0001**	3.9% (3.8,3.9)	0.10
(e.g., ln(52) = 3.95)	4	**4.4% (4.4,4.5)**		**4.0% (3.9,4.1)**		**4.5% (4.4,4.6)**		4.0% (3.9,4.1)	
**First Person Singular**	1	**4.3% (4.0,4.6)**	**<.0001**	**3.8% (3.4,4.3)**	**0.0003**	**4.2% (3.7,4.7)**	**<.0001**	3.6% (3.0,4.2)	0.12
(e.g., I, me, mine)	4	**2.4% (2.0,2.7)**		**2.7% (2.3,3.2)**		**2.6% (2.1,3.1)**		2.9% (2.3,3.5)	
**First Person Plural**	1	2.5% (2.2,2.7)	0.42	2.3% (1.9,2.6)	0.04	2.3% (2.0,2.7)	0.12	2.3% (1.9,2.8)	0.64
(e.g., we, us, our)	4	2.6% (2.3,2.9)		2.8% (2.4,3.1)		2.8% (2.4,3.2)		2.5% (2.0,2.9)	
**Past Focus**	1	2.6% (2.6,2.6)	0.02	2.8% (2.5,3.1)	0.03	2.3% (2.0,2.6)	0.11	2.8% (2.4,3.2)	0.40
(e.g., ago, did, talked)	4	2.6% (2.6,2.7)		2.3% (2.0,2.7)		1.9% (1.6,2.2)		2.5% (2.1,3.0)	
**Present Focus (log)**	1	2.2% (2.0,2.4)	0.63	2.5% (2.5,2.6)	0.004	2.6% (2.5,2.6)	0.72	2.6% (2.5,2.6)	0.20
(e.g., today, is, now)	4	2.1% (1.9,2.3)		2.6% (2.6,2.7)		2.6% (2.5,2.7)		2.6% (2.6,2.7)	
**Future Focus**	1	-	-	-	-	-	-	-	-
(e.g., may, will, soon)	4	-		-		-		-	
**Positive Emotion**	1	**4.3% (4.0,4.6)**	**<.0001**	3.4% (3.0,3.7)	0.46	4.2% (3.8,4.6)	0.07	3.2% (2.7,3.6)	0.27
(e.g, love, nice, sweet)	4	**3.3% (3.0,3.6)**		3.2% (2.8,3.5)		3.6% (3.2,4.1)		2.8% (2.3,3.3)	
**Negative Emotion**	1	**1.7% (1.5,1.8)**	**<.0001**	**1.7% (1.4,1.9)**	**<.0001**	**1.4% (1.2,1.7)**	**<.0001**	2.0% (1.6,2.3)	0.09
(e.g, hurt, ugly, nasty)	4	**2.4% (2.2,2.6)**		**2.6% (2.3,2.8)**		**2.4% (2.1,2.7)**		2.4% (2.1,2.8)	
**Sad**	1	-	-	-	-	-	-	-	-
(e.g, worried, fearful)	4	-		-		-		-	
**Anger**	1	-	-	-	-	-	-	-	-
(e.g., hate, kill, annoyed)	4	-		-		-		-	
**Anxiety**	1	-	-	-	-	-	-	-	-
(e.g., worried, fearful)	4	-		-		-		-	
**Assent**	1	-	-	-	-	-	-	-	-
(e.g., agree, OK, yes)	4	-		-		-		-	
**Social**	1	8.8% (8.4,9.2)	0.69	7.4% (6.8,7.9)	0.03	8.6% (8.0,9.2)	0.91	7.4% (6.6,8.1)	0.32
(e.g., mate, talk, they)	4	8.6% (8.2,9.1)		8.3% (7.7,8.8)		8.7% (8.0,9.3)		7.9% (7.1,8.7)	
**Family**	1	-	-	-	-	-	-	-	-
(e.g., daughter, aunt)	4	-		-		-		-	
**Friends**	1	-	-	-	-	-	-	-	-
(e.g., buddy, neighbor)	4	-		-		-		-	
**Power**	1	**4.3% (4.0,4.6)**	**<.0001**	**3.9% (3.4,4.3)**	**<.0001**	**4.3% (3.9,4.8)**	**0.0002**	4.4% (3.8,5.0)	0.05
(e.g, own, order, allow)	4	**5.7% (5.4,6.0)**		**5.2% (4.7,5.6)**		**5.6% (5.1,6.1)**		5.2% (4.6,5.8)	

Note: For comments with WC≥20, demographic data was missing for 1,180 (56.0%) comments in 2019 and 180 (11.2%) comments in 2020.

App. Table 2. Frequencies of all Linguistic Categories in Healthcare Worker Comments Across Levels of Emotional Exhaustion (EE) Controlling for Word Count and Demographics

**Linguistic Category** (example words)	**Quartile** 1: min EE 4: max EE	Same-Year Comparisons				Across-Year Comparisons			
		**2019 Comments & 2019 Emotional Exhaustion**		**2020 Comments & 2020 Emotional Exhaustion**		**2019 Comments & 2020 EE**		**2019 EE & 2020 Comments**	
		Percent (95% CI)	p	Percent (95% CI)	p	Percent (95% CI)	p	Percent (95% CI)	p
**Word Count (log)**	1	**4.0% (3.9,4.1)**	**<.0001**	**3.8% (3.7,3.8)**	**<.0001**	**4.0% (3.9,4.1)**	**<.0001**	3.9% (3.8,3.9)	0.11
(e.g., ln(52) = 3.95)	4	**4.5% (4.4,4.6)**		**4.0% (3.9,4.1)**		**4.5% (4.4,4.6)**		4.0% (3.9,4.1)	
**First Person Singular**	1	**4.9% (4.4,5.3)**	**<.0001**	**3.8% (3.4,4.3)**	**0.0006**	**4.2% (3.7,4.7)**	**<.0001**	3.6% (3.0,4.1)	0.20
(e.g., I, me, mine)	4	**2.8% (2.2,3.3)**		**2.7% (2.3,3.2)**		**2.6% (2.1,3.2)**		3.0% (2.4,3.6)	
**First Person Plural**	1	2.5% (2.1,2.9)	0.07	2.3% (2.0,2.6)	0.08	2.4% (2.0,2.7)	0.21	2.3% (1.9,2.8)	0.66
(e.g., we, us, our)	4	3.0% (2.6,3.4)		2.7% (2.4,3.1)		2.7% (2.3,3.1)		2.5% (2.0,2.9)	
**Past Focus**	1	2.1% (1.8,2.4)	0.17	2.8% (2.5,3.1)	0.07	2.2% (1.9,2.5)	0.20	2.8% (2.4,3.2)	0.52
(e.g., ago, did, talked)	4	1.8% (1.5,2.1)		2.4% (2.0,2.7)		1.9% (1.6,2.3)		2.6% (2.1,3.0)	
**Present Focus (log)**	1	2.6% (2.5,2.6)	0.05	2.5% (2.5,2.6)	0.004	2.6% (2.5,2.6)	0.46	2.6% (2.5,2.6)	0.17
(e.g., today, is, now)	4	2.7% (2.6,2.7)		2.6% (2.6,2.7)		2.6% (2.6,2.7)		2.6% (2.6,2.7)	
**Future Focus**	1	-	-	-	-	-	-	-	-
(e.g., may, will, soon)	4	-		-		-		-	
**Positive Emotion**	1	**4.5% (4.1,4.9)**	**0.0003**	3.3% (3.0,3.7)	0.48	4.2% (3.8,4.6)	0.05	3.2% (2.7,3.6)	0.28
(e.g, love, nice, sweet)	4	**3.3% (2.8,3.8)**		3.2% (2.8,3.5)		3.6% (3.1,4.1)		2.8% (2.4,3.3)	
**Negative Emotion**	1	1.5% (1.3,1.8)	0.001	**1.6% (1.4,1.9)**	**<.0001**	**1.5% (1.2,1.8)**	**<.0001**	2.0% (1.6,2.3)	0.09
(e.g, hurt, ugly, nasty)	4	2.3% (1.9,2.6)		**2.6% (2.3,2.8)**		**2.4% (2.1,2.7)**		2.4% (2.1,2.8)	
**Sad**	1	-	-	-	-	-	-	-	-
(e.g, worried, fearful)	4	-		-		-		-	
**Anger**	1	-	-	-	-	-	-	-	-
(e.g., hate, kill, annoyed)	4	-		-		-		-	
**Anxiety**	1	-	-	-	-	-	-	-	-
(e.g., worried, fearful)	4	-		-		-		-	
**Assent**	1	-	-	-	-	-	-	-	-
(e.g., agree, OK, yes)	4	-		-		-		-	
**Social**	1	9.0% (8.4,9.6)	0.67	7.4% (6.8,7.9)	0.05	8.6% (8.0,9.2)	0.92	7.3% (6.6,8.1)	0.30
(e.g., mate, talk, they)	4	8.8% (8.1,9.5)		8.2% (7.6,8.8)		8.6% (7.9,9.3)		7.9% (7.1,8.7)	
**Family**	1	-	-	-	-	-	-	-	-
(e.g., daughter, aunt)	4	-		-		-		-	
**Friends**	1	-	-	-	-	-	-	-	-
(e.g., buddy, neighbor)	4	-		-		-		-	
**Power**	1	**4.1% (3.6,4.5)**	**<.0001**	**3.8% (3.4,4.3)**	**<.0001**	**4.4% (3.9,4.9)**	**0.0010**	4.4% (3.9,5.0)	0.07
(e.g, own, order, allow)	4	**6.0% (5.5,6.5)**		**5.1% (4.7,5.6)**		**5.6% (5.1,6.1)**		5.2% (4.6,5.8)	

App. Table 3. Comprehensive survey respondent demographics.

Demographics	All Responses N=17,054	2019 No Comments N=8,025	2019 Excluded Comments (WC:0-19) N=1,150	2019 Comments N=2,109	2020 No Comments N=6,977	2020 Excluded Comments (WC: 1-19) N=1,643	2020 Comments N=1,603
**Age (μ; 95%CI)**	45.5 (45.3,45.7)	45.2 (44.9,45.5)	48.6 (47.4,49.7)	46.7 (45.9,47.4)	45.2 (44.9,45.5)	46.4 (45.8,47.1)	46 (45.4,46.6)
**Gender**							
Female	8,215 (83.3%)	7,057 (83.1%)	358 (81.4%)	800 (86.1%)	5,749 (83.6%)	1,228 (78.6%)	1,238 (87.0%)
Male	1,648 (16.7%)	1,437 (16.9%)	82 (18.6%)	129 (13.9%)	1,128 (16.4%)	335 (21.4%)	185 (13.0%)
**Race**							
Two or More	94 (1.0%)	80 (0.9%)	3 (0.7%)	11 (1.2%)	71 (1.0%)	10 (0.6%)	13 (0.9%)
American Indian or Alaska Native	47 (0.5%)	38 (0.4%)	2 (0.5%)	7 (0.8%)	35 (0.5%)	5 (0.3%)	7 (0.5%)
Asian	192 (1.9%)	170 (2.0%)	10 (2.3%)	12 (1.3%)	133 (1.9%)	35 (2.2%)	24 (1.7%)
Black	578 (5.9%)	499 (5.9%)	33 (7.5%)	46 (5.0%)	410 (6.0%)	122 (7.8%)	46 (3.2%)
Hispanic/Latino	181 (1.8%)	145 (1.7%)	10 (2.3%)	26 (2.8%)	126 (1.8%)	30 (1.9%)	25 (1.8%)
Pacific Islander or Native Hawaiian	8 (0.1%)	6 (0.1%)	1 (0.2%)	1 (0.1%)	4 (0.1%)	3 (0.2%)	1 (0.1%)
White	8,706 (88.3%)	7,499 (88.3%)	381 (86.6%)	826 (88.9%)	6,055 (88.0%)	1,346 (86.1%)	1,305 (91.7%)
Other	41 (0.4%)	41 (0.5%)	0 (0%)	0 (0%)	31 (0.5%)	9 (0.6%)	1 (0.1%)
**Is a Registered Nurse? (Y/N)**	2,173 (22.0%)	1,765 (20.8%)	118 (26.8%)	290 (31.2%)	1,386 (20.2%)	385 (24.6%)	402 (28.3%)
**Is a Supervisor? (Y/N)**	927 (9.4%)	7,678 (90.4%)	401 (91.1%)	857 (92.2%)	6,137 (89.2%)	1,457 (93.2%)	1,342 (94.3%)
**Job Classification**							
Administrative Support Workers	1,860 (18.9%)	1,615 (19.0%)	67 (15.2%)	178 (19.2%)	1,337 (19.4%)	276 (17.7%)	247 (17.4%)
Craft Workers	78 (0.8%)	72 (0.8%)	3 (0.7%)	3 (0.3%)	56 (0.8%)	20 (1.3%)	2 (0.1%)
Executive/Sr Level Officials	112 (1.1%)	104 (1.2%)	2 (0.5%)	6 (0.6%)	101 (1.5%)	9 (0.6%)	2 (0.1%)
First/Mid-Level Officials	928 (9.4%)	792 (9.3%)	51 (11.6%)	85 (9.1%)	721 (10.5%)	120 (7.7%)	87 (6.1%)
Professionals	3,818 (38.7%)	3,185 (37.5%)	179 (40.7%)	454 (48.9%)	2,588 (37.6%)	591 (37.8%)	639 (44.9%)
Service Workers	1,212 (12.3%)	1,045 (12.3%)	70 (15.9%)	97 (10.4%)	789 (11.5%)	270 (17.3%)	153 (10.8%)
Technicians	1,191 (12.1%)	1,018 (12.0%)	67 (15.2%)	106 (11.4%)	805 (11.7%)	197 (12.6%)	189 (13.3%)
**2019 Emotional Exhaustion (μ; 95%CI)**	45.3 (44.9,45.8)	42.0 (41.5,42.5)	43.4 (41.6,45.2)	59.2 (57.9,60.6)	45.0 (44.4,45.5)	43.6 (42.1,45.1)	52.0 (50.4,53.5)
**2020 Emotional Exhaustion (μ; 95%CI)**	44.8 (44.4,45.3)	43.5 (43.0,44.0)	46.1 (44.2,48.0)	57.0 (55.6,58.3)	42.2 (41.7,42.8)	43.0 (41.4,44.5)	60.2 (58.6,61.8)

Note: All Responses includes the demographics for all survey responses, regardless of if they contained a comment (N=17,054). In total, 2019 (N=2,109) and 2020 (N=1,606) responses had comments with a word count (WC) of 20 or more. Demographic data was available for some of these responses in 2019 (n=929) and 2020 (n=1,423). The continuous variables age & emotional exhaustion are reported as the mean (with 95% confidence intervals). The categorical variables are reported as the number of responses (with percent makeup of the demographic category). For EE scores in All Responses, there are 39 (0.3%) missing responses for 2019 and 322 (3.0%) missing responses for 2020. In 2020, 260 respondents commented without filling out the remaining survey items. The 2019 & 2020 comments each have less than 5 missing EE scores per Comment-EE comparison.

App. Table 4. Sample size and emotional exhaustion (EE) range of each EE quartile for all study comparisons controlling only for word count

**Emotional Exhaustion Year**	**Word Count Year**	**Total Sample Size**	**Quartile 1**		**Quartile 2**		**Quartile 3**		**Quartile 4**	
			N	EE Range	N	EE Range	N	EE Range	N	EE Range
2019	2019	2101	570	[0,35)	541	[35,65)	486	[65,85)	504	[85,100]
	2020	924	258	[0,35)	231	[35,60)	225	[60,85)	210	[85,100]
2020	2019	770	200	[0,25)	212	[25,50)	173	[55,75)	185	[75,100]
	2020	1418	359	[0,35)	385	[35,55)	338	[65,90)	336	[90,100]
