# Supplementary figures and images for: The language of healthcare worker emotional exhaustion: A linguistic analysis of longitudinal survey
Source: Front Psychiatry. 2022 Dec 16;13:1044378. doi: 10.3389/fpsyt.2022.1044378 (PMC9800594; doi:10.3389/fpsyt.2022.1044378)

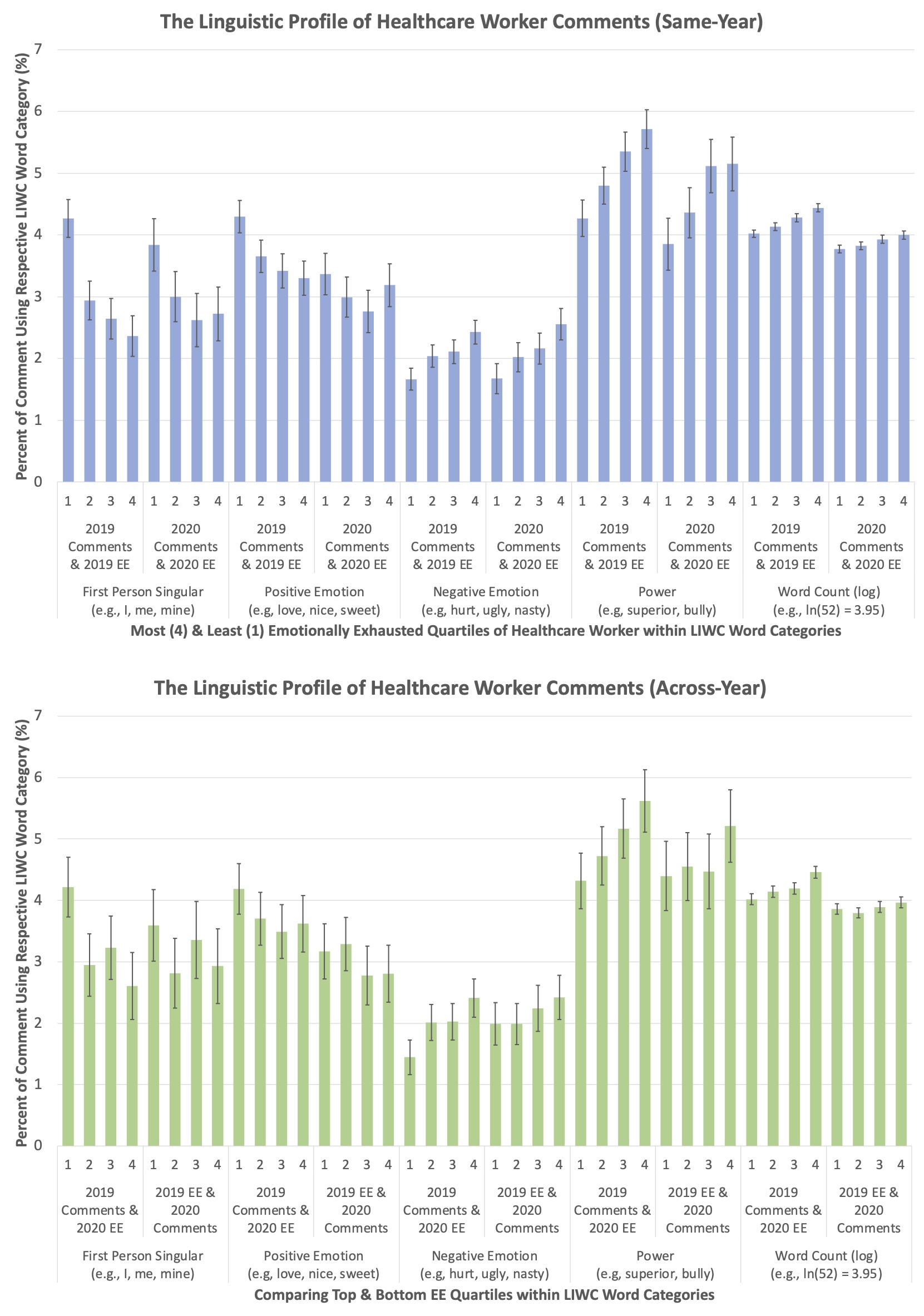

Supplement: Supplementary file 2 [file Image_1.JPEG]

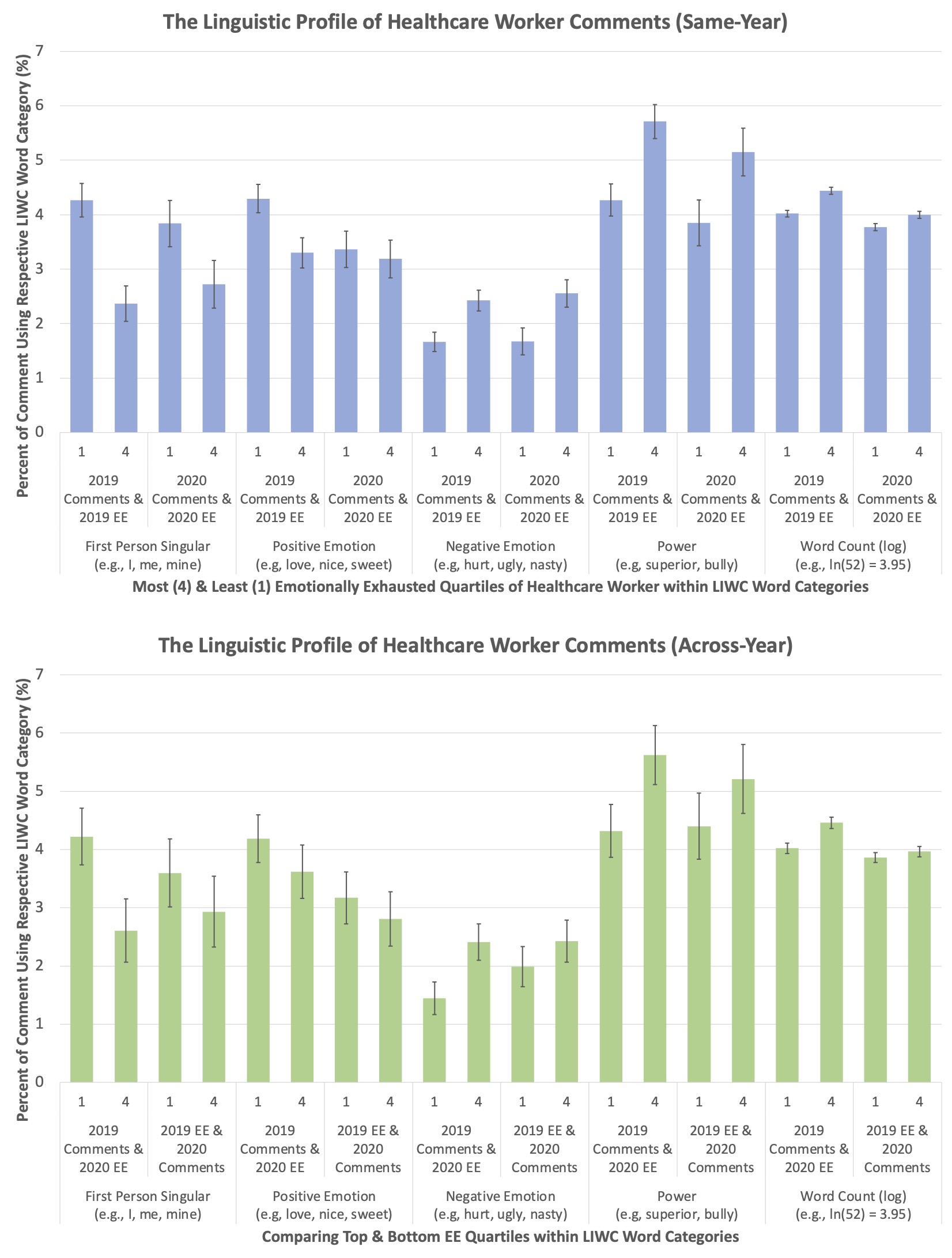

Supplement: Supplementary file 3 [file Image_2.JPEG]
